# Role of ethambutol and rifampicin in the treatment of *Mycobacterium avium* complex pulmonary disease

**DOI:** 10.1186/s12890-019-0982-8

**Published:** 2019-11-11

**Authors:** Hyung-Jun Kim, Jong Sik Lee, Nakwon Kwak, Jaeyoung Cho, Chang-Hoon Lee, Sung Koo Han, Jae-Joon Yim

**Affiliations:** 1 Division of Pulmonary and Critical Care Medicine, Department of Internal Medicine, Seoul National University Hospital, 101 Daehak-ro Jongno-gu, Seoul, 03080 Republic of Korea; 2 Division of Pulmonary Medicine, Department of Internal Medicine, Mediplex Sejong Hospital, 20 Gyeyangmunhwa-ro Gyeyang-gu, Incheon, 21080 Republic of Korea

**Keywords:** *Mycobacterium avium* complex pulmonary disease, Ethambutol, Rifampicin

## Abstract

**Background:**

A three-drug regimen (macrolide, ethambutol, and rifampicin) is recommended for the treatment of *Mycobacterium avium* complex pulmonary disease (MAC-PD). Although macrolide has proven efficacy, the role of ethambutol and rifampicin in patients without acquired immune deficiency syndrome is not proven with clinical studies. We aimed to clarify the roles of ethambutol and rifampicin in the treatment of MAC-PD.

**Methods:**

Patients treated for MAC-PD between March 1st, 2009 and October 31st, 2018 were reviewed retrospectively. Rates of culture conversion, microbiological cure, treatment failure, and recurrence were compared according to the maintenance (≥6 months) of ethambutol or rifampicin with macrolide.

**Results:**

Among the 237 patients, 122 (51.5%) maintained ethambutol and rifampicin with macrolide, 58 (24.5%) maintained ethambutol and macrolide, 32 (13.5%) maintained rifampicin and macrolide, and 25 (10.6%) maintained macrolide only. Culture conversion was reached for 190/237 (80.2%) patients and microbiological cure was achieved for 129/177 (72.9%) who completed the treatment. Treatment failure despite ≥12 months of treatment was observed in 66/204 (32.4%), and recurrence was identified in 16/129 (12.4%) who achieved microbiological cure. Compared with maintenance of macrolide only, maintenance of ethambutol, rifampicin or both with macrolide were associated with higher odds of culture conversion [odds ratio (OR), 95% confidence interval (CI): 18.06, 3.67–88.92; 15.82, 2.38–105.33; and 17.12, 3.93–74.60, respectively]. Higher odds of microbiological cure were associated with maintenance of both ethambutol and rifampicin with macrolide (OR, 95% CI: 5.74, 1.54–21.42) and macrolide and ethambutol (OR, 95% CI: 5.12, 1.72–15.24) but not macrolide and rifampicin. Maintenance of both ethambutol and rifampicin with macrolide was associated with lower odds of treatment failure (OR, 95% CI: 0.09, 0.01–0.53) compared with macrolide only, while maintenance of one of these with macrolide was not. Maintenance of both ethambutol and rifampicin or one of these with macrolide did not decrease the probability of recurrence when compared with macrolide only.

**Conclusions:**

Maintenance (≥6 months) of ethambutol and rifampicin with macrolide was associated with the most favorable treatment outcomes among patients with MAC-PD. Given the association between ongoing ethambutol use and microbiological cure, clinicians should maintain ethambutol unless definite adverse events develop.

## Background

The prevalence of nontuberculous mycobacterial (NTM) pulmonary disease is increasing globally, with the most common pathogen being *Mycobacterium avium* complex (MAC) species [1–3]. MAC is a ubiquitous organism commonly found in water, dirt, and soil but can cause serious pulmonary diseases in some people. *M. avium* and *M. intracellulare* are the most well-known pathogens, but other MAC such as *M. arosiense*, *M. bouchedurhonense*, *M. chimaera*, *M. colombiense*, *M. marseillense*, *M. timonense*, *M. vulneris*, and *M. yongonense* can also cause pulmonary diseases [4].

Treatment for MAC pulmonary disease (MAC-PD) requires consideration of clinical and/or radiographical deterioration and usually comprises a multidrug regimen including macrolide, ethambutol, and rifampicin, with varying success [5]. A recent systematic review reported that the three-drug regimen (macrolide, ethambutol, and rifampicin) with occasional use of an injectable agent could cure MAC-PD in 57% of patients [6].

The recommendation of such a combination is based on previous studies about disseminated MAC disease in acquired immune deficiency syndrome (AIDS) patients [7]. The efficacy of macrolide among MAC-PD patients is well established, but macrolide monotherapy can induce microbiological resistance [8]. Therefore, companion drugs such as ethambutol or rifampicin are used. Nevertheless, the individual effectiveness of these two drugs in patients without AIDS is not proven with clinical studies. Considering the long-term use of medication and possible adverse events, recognizing the most efficacious accompanying drug is necessary.

The aim of this study was to elucidate the roles of ethambutol and rifampicin in the treatment of MAC-PD in adult patients.

## Methods

### Study population

Patients aged 19 years or older in whom treatment for MAC-PD was initiated between March 1st, 2009 and October 31st, 2018 at Seoul National University Hospital were included. Diagnosis of MAC-PD was based on the 2007 American Thoracic Society and the Infectious Diseases Society of America guideline [5] and the 2017 British Thoracic Society guideline [9]. Some patients were included in our previous reports [10, 11].

The study was conducted in accordance with the amended Declaration of Helsinki and was approved by the institutional review board of Seoul National University Hospital (protocol number: H-1705-017-851). Informed consent was waived because of the retrospective design of the study, and the information of each patient was anonymized prior to analyses.

### Clinical, microbiological, and radiographic evaluation

Clinical, microbiological, and radiographic information were collected retrospectively. Cultures were grown in both solid Ogawa media and the BACTEC MGIT 960 system, and isolated NTM were identified into species [4]. Identification was performed based on sequence analysis of the 16S rRNA and *rpoB* gene using the algorithm described in the Clinical and Laboratory Standards Institute guidelines MM18-A [12]. Once the NTM was identified as MAC, a drug susceptibility test for clarithromycin was performed. The minimal inhibitory concentration (MIC) test was performed at the Korean Institute of Tuberculosis, and was determined using the broth microdilution method in accordance with the Clinical and Laboratory Standards Institute guidelines [12]. Isolates were considered susceptible if the MIC of clarithromycin was ≤8 μg/mL, resistant if ≥32 μg/mL, and intermediate if 16 μg/mL on Mueller–Hinton agar. Chest computed tomography findings were categorized as the nodular bronchiectatic form when bilateral bronchiectasis and cellular bronchiolitis were mainly present, and the upper lobe cavitary form when cavities in the upper lobes were observed [13].

### Treatment and follow-up

The treatment regimen was mainly based on the 2007 American Thoracic Society and the Infectious Diseases Society of America guidelines, but was customized by the attending physician according to each patient’s condition [5]. Macrolide was included in every regimen, and ethambutol and rifampicin were considered as companion drugs. The companion drugs were omitted or stopped when they were considered clinically inappropriate; for example, ethambutol was omitted or stopped if a patient’s visual acuity deteriorated, and rifampicin was omitted or stopped when a patient had underlying liver diseases or newly developed hepatic dysfunction. Injectable drugs including streptomycin or amikacin were considered when the MAC-PD was extensive or refractory to other oral agents.

Adverse reactions to medications were recorded by the physician. Subjective events such as decreased visual acuity, deteriorating hearing, and nausea were either self-reported or enquired after by the attending physician. If patients had symptoms suggesting adverse drug reactions, they were referred to relevant specialists. The presumed offending drug was discontinued when clinically necessary.

### Definitions

Maintenance of ethambutol or rifampicin was defined as more than 6 months of use as adopted in a previous study [14]. Clinical outcomes encompassing culture conversion, microbiological cure, treatment failure, and recurrence were defined among patients with ≥3 follow-up sputum culture studies. ‘Culture conversion’ was defined as three consecutive negative sputum culture results after treatment [15, 16]. The first date of culture-negative sputum specimen collection was defined as the day of culture conversion. ‘Microbiological cure’ was defined as the maintenance of negative culture conversion at the point of treatment completion [16]. ‘Treatment failure’ was defined as the persistence or re-emergence of multiple positive cultures for the causative species of NTM from respiratory samples after ≥12 months of antimycobacterial treatment while the patient was still on treatment [16]. ‘Recurrence’ was defined as the re-emergence of causative species of NTM with ≥2 positive cultures from sputum after achieving a microbiological cure [15, 16]. The first day of culture-positive sputum specimen collection was considered as the day of recurrence.

### Statistical analysis

Values were organized as numbers (percentages) for dichotomous variables, and medians with interquartile range (IQR) for continuous variables. Logistic regression analysis was performed to seek potential predictors for culture conversion, microbiological cure, and treatment failure. Odds ratios (ORs) with 95% confidence intervals (CI) for each candidate predictor were calculated, and candidates with a *p* < 0.05 were included in the multivariable models. Kaplan-Meier curves were drawn with log-rank tests to compare probabilities of initial culture conversion and recurrence after treatment success, according to maintenance of the ethambutol and rifampicin. All statistical analyses and figure drawings were performed using Stata ver. 13.0 (Stata Corp., College Station, TX).

## Results

### Baseline characteristics

During the 9-year study period, 331 patients with MAC-PD initiated treatment. The following patients were excluded from analysis: 28 patients with short (< 6 months) duration of treatment at the time point of data collection, 26 patients who were previously treated for MAC-PD, 16 patients without ≥3 follow-up cultures, 16 patients with poor compliance to prescriptions (> 1-month omission of medication during the treatment period), and 8 patients with clarithromycin-resistant MAC infection. The remaining 237 patients were included for further analysis.

Of the 237 patients treated for MAC-PD, the median age was 64 years (IQR: 57–73) and 147 (62.0%) were female. All patients underwent baseline evaluation for the presence of human immunodeficiency virus antibody, all of which produced a negative result. *M. avium* (50.6%) and *M. intracellulare* (47.7%) were the main species detected. Among the 190 patients who underwent drug susceptibility testing for clarithromycin, the MIC were 8 μg/mL (2 patients), 4 μg/mL (18 patients), 2 μg/mL (69 patients), 1 μg/mL (78 patients), and ≤ 0.5 μg/mL (23 patients). Upon diagnosis, 81 (34.2%) patients had positive acid-fast bacilli smear results. The nodular bronchiectatic form was the dominant radiographic pattern (79.8%).

Among the 237 patients, 122 (51.5%) maintained treatment (≥6 months) with all three drugs, 58 (24.5%) maintained ethambutol and macrolide, 32 (13.5%) maintained rifampicin and macrolide, and 25 (10.6%) maintained macrolide only. Patients who maintained all three agents tended to be younger (*p* = 0.003) and had a higher proportion of infections with *M. avium* (*p* = 0.018) compared with the other groups of patients (Table 1).

**Table 1:** Baseline characteristics patients according to the maintenance (≥6mo) of antimycobacterial agents

Variables	Macrolide only *n* = 25	Macrolide and ethambutol *n* = 58	Macrolide and rifampicin *n* = 32	Macrolide, ethambutol, and rifampicin *n* = 122	*P*
Age (years)	71 (65–80)	66 (58–75)	66 (56–73)	62 (56–70)	0.003
BMI (kg/m^2^)	20 (18–22)	21 (18–22)	20 (19–23)	21 (19–22)	0.649
Sex, female	11 (44.0%)	34 (58.6%)	18 (56.3%)	84 (68.9%)	0.086
Smoking history					0.444
Never smoker	12 (48.0%)	35 (60.3%)	21 (65.6%)	82 (67.2%)	
Former smoker	9 (36.0%)	14 (24.1%)	5 (15.6%)	21 (17.2%)	
Current smoker	1 (4.0%)	3 (5.2%)	1 (3.1%)	2 (1.6%)	
Comorbidities					
Asthma	2 (8.0%)	0 (0.0%)	1 (3.1%)	2 (1.6%)	0.093
COPD	2 (8.0%)	0 (0.0%)	0 (0.0%)	2 (1.6%)	0.116
History of tuberculosis	11 (44.0%)	17 (29.3%)	7 (21.9%)	26 (21.3%)	0.100
Drug use					
Immunomodulatory drugs	2 (8.0%)	0 (0.0%)	0 (0.0%)	6 (4.9%)	0.112
Steroid	1 (4.0%)	1 (1.7%)	1 (3.1%)	3 (2.5%)	0.799
MAC species					0.018
*M. avium*	9 (36.0%)	24 (41.4%)	12 (37.5%)	75 (61.5%)	
*M. intracellulare*	15 (60.0%)	33 (56.9%)	20 (62.5%)	45 (36.9%)	
*M. chimaera*	1 (4.0%)	1 (1.7%)	0 (0.0%)	1 (0.8%)	
*M. columbiense*	0 (0.0%)	0 (0.0%)	0 (0.0%)	1 (0.8%)	
Smear positivity at diagnosis	14 (56.0%)	19 (32.8%)	10 (31.3%)	38 (31.2%)	0.113
Radiographic pattern					0.847
Nodular bronchiectatic	19 (76.0%)	45 (77.6%)	25 (78.1%)	100 (82.0%)	
Upper lobe cavitary	6 (24.0%)	13 (22.4%)	7 (21.9%)	22 (18.0%)	
Pulmonary function tests					
FVC (% predicted)	79 (63–86)	88 (75–97)	90 (74–96)	89 (77–99)	0.086
FEV_1_ (% predicted)	92 (64–99)	94 (79–104)	90 (80–108)	95 (83–109)	0.582
FEV_1_/FVC (%)	80 (64–89)	78 (72–83)	79 (70–87)	77 (71–84)	0.928

Values are presented as number (percentage) or median (interquartile range)

*Abbreviations*: *BMI* body mass index, *COPD* chronic obstructive pulmonary disease, *MAC Mycobacterium avium* complex, *FVC* forced vital capacity, *FEV*_*1*_ forced expiratory volume in 1 s

### Treatment regimen

After initiation of treatment, patients were followed on 4- to 8-week intervals and sputum specimens were requested to be submitted for acid-fast bacilli smears and mycobacterial cultures on each visit. Median treatment duration was 18.6 months (IQR: 16.3–24.3). Azithromycin was prescribed in 183 patients (77.2%) and clarithromycin in 54 (22.8%). For 224 (94.5%) patients, ethambutol was received for a median of 16.3 (IQR: 7.7–21.0) months, and 180 (80.4%) maintained the drug for longer than 6 months. For 179 (75.9%) patients, rifampicin was received for a median of 18.2 (IQR: 11.1–23.8) months, and 154 (86.0%) maintained it for longer than 6 months (Table 2).

**Table 2:** Treatment regimen and outcomes of 237 patients with *Mycobacterium avium* complex lung disease

Categories	Variables	Values
Drug use	Macrolide	237 (100.0%)
	Duration, months	18.6 (16.3–24.3)
	Ethambutol	224 (94.5%)
	Maintenance (≥6 months)	180 (80.4%)
	Duration, for patients with maintenance, months	18.0 (12.1–22.0)
	Duration, for patients without maintenance, months	1.2 (0.0–3.2)
	Rifampicin	179 (75.9%)
	Maintenance (≥6 months)	154 (86.0%)
	Duration, for patients with maintenance, months	18.7 (16.7–24.3)
	Duration, for patients without maintenance, months	0.0 (0.0–0.5)
Outcomes	Culture conversion^a^	190/237 (80.2%)
	Microbiological cure^b^	129/177 (72.9%)
	Treatment failure^c^	66/204 (32.4%)
	Recurrence^d^	16/129 (12.4%)

Values are presented as number (percentage) or median (interquartile range)

^a^At least three consecutive negative results for sputum culture after the start of treatment. The first day of a negative result was considered the date of culture conversion

^b^Maintenance of negative culture conversion until the end of treatment. Assessed among patients who stopped taking antibiotics

^c^Re-emergence of multiple positive cultures or persistence with the causative species from respiratory samples after ≥12 months of antimycobacterial treatment, while the patient is still on treatment. Assessed among patients with ≥12 months of antimycobacterial treatment

^d^The re-emergence of at least 2 positive cultures with the same species from sputum after cessation of antimycobacterial treatment. Assessed among patients with microbiological cure

Injectable agents were administered to 24 patients (10.1%) and these included streptomycin (18 patients) and amikacin (6 patient) (Table 3). A daily regimen was prescribed for 146 patients (61.6%), while 91 (38.4%) started a three-times-weekly regimen. The median dosage of ethambutol was 15.4 mg/kg for the daily regimen and 22.6 mg/kg for the three-times-weekly regimen. Other detailed dosage information is described in (Additional file 1: Table S1).

**Table 3:** Detailed description of treatment regimens prescribed in 237 patients

Treatment regimens	Values
Macrolide + Ethambutol + Rifampicin	131 (55.3%)
Macrolide + Ethambutol	43 (18.1%)
Macrolide + Ethambutol + Rifampicin + Clofazimine	11 (4.6%)
Macrolide + Ethambutol + Rifampicin + Injection drug	10 (4.2%)
Macrolide + Ethambutol + Rifampicin + Quinolone	7 (3.0%)
Macrolide + Ethambutol + Clofazimine	6 (2.5%)
Macrolide + Rifampicin	6 (2.5%)
Macrolide	4 (1.7%)
Macrolide + Ethambutol + Injection drug	3 (1.3%)
Macrolide + Rifampicin + Injection drug	2 (0.8%)
Macrolide + Ethambutol + Rifampicin + Quinolone + Injection drug	2 (0.8%)
Macrolide + Rifampicin + Clofazimine	1 (0.4%)
Macrolide + Ethambutol + Clofazimine + Quinolone	1 (0.4%)
Macrolide + Ethambutol + Clofazimine + Injection drug	1 (0.4%)
Macrolide + Ethambutol + Quinolone + Injection drug	1 (0.4%)
Macrolide + Ethambutol + Rifampicin + Injection drug/Inhaled amikacin	1 (0.4%)
Macrolide + Ethambutol + Rifampicin + Quinolone + Inhaled amikacin	1 (0.4%)
Macrolide + Ethambutol + Rifampicin + Cycloserine + Pyrazinamide	1 (0.4%)
Macrolide + Ethambutol + Rifampicin + Amoxicillin/clavulanate	1 (0.4%)
Macrolide + Ethambutol + Rifampicin + Clofazimine + Injection drug/Inhaled amikacin	1 (0.4%)
Macrolide + Ethambutol + Rifampicin + Clofazimine + Quinolone + Injection drug	1 (0.4%)
Macrolide + Ethambutol + Rifampicin + Quinolone + Amoxicillin/clavulanate	1 (0.4%)
Macrolide + Ethambutol + Rifampicin + Quinolone + Injection drug/Inhaled amikacin	1 (0.4%)

Values are presented as numbers (%). Includes drugs used after the diagnosis of *Mycobacterium avium* complex lung disease

### Treatment outcomes

Culture conversion was achieved in 190 out of 237 (80.2%) patients after a median of 1.7 (IQR: 0.5–4.7) months of treatment, and a microbiological cure was achieved in 129 out of 177 (72.9%) patients who completed treatment. Of the 60 patients who did not complete treatment, 58 were still on active treatment, and 2 were lost to follow-up. The treatment failure rate was 32.4% (66 patients) among 204 patients with ≥12 months of treatment. Among the 129 patients who achieved a microbiological cure, 16 patients (12.4%) experienced recurrence with the same species of MAC (Table 2).

**Fig. 1:**
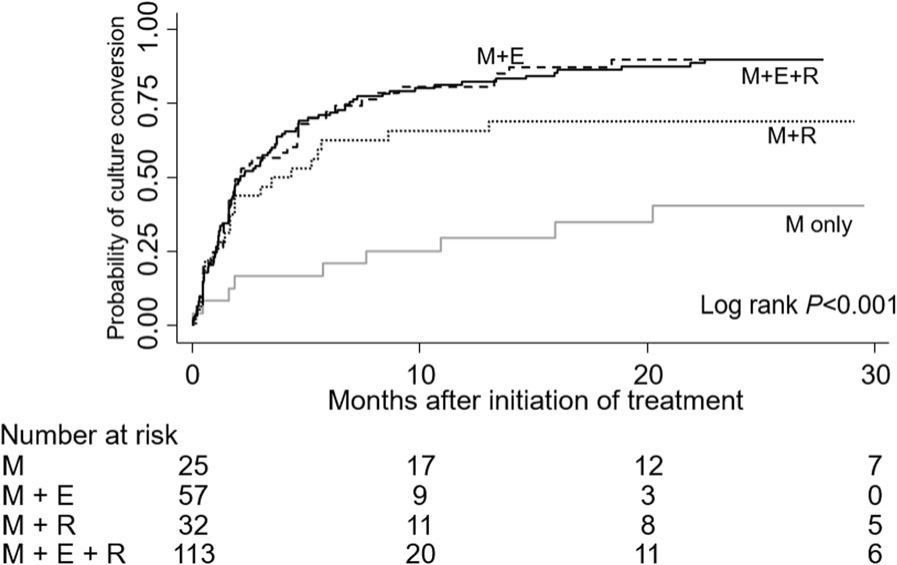
Probability of culture conversion according to the maintenance (≥6 months) of ethambutol and rifampicin. Abbreviations: M, macrolide; E, ethambutol; R, rifampicin

Patients who maintained (≥6 months) treatment with either ethambutol, rifampicin or both with macrolide had a higher probability of culture conversion compared with maintenance of macrolide only (log-rank *p* < 0.001) (Fig. 1). Maintenance of ethambutol and rifampicin with macrolide (adjusted OR 17.12 with 95% CI 3.93–74.60), ethambutol and macrolide (adjusted OR 18.06 with 95% CI 3.67–88.92), and rifampicin and macrolide (adjusted OR 15.82 with 95% CI 2.38–105.33) showed higher odds for culture conversion compared with the macrolide only group. (Table 4).

**Table 4:** Predictors for culture conversion among treated *Mycobacterium avium* complex pulmonary disease patients

Variables	Unadjusted OR (95% CI)	*P*	Adjusted OR (95% CI)	*P*
Age (years)	0.95 (0.92–0.98)	0.003	1.01 (0.96–1.06)	0.680
BMI (kg/m^2^)	1.23 (1.06–1.41)	0.005	1.21 (1.02–1.44)	0.031
Sex, female	2.72 (1.42–5.23)	0.003	1.43 (0.55–3.74)	0.468
History of tuberculosis	0.53 (0.27–1.05)	0.070		
MAC species				
*M. avium*	Reference		Reference	
*M. intracellulare*	0.26 (0.13–0.53)	< 0.001	0.29 (0.10–0.84)	0.022
Smear positivity at diagnosis	0.18 (0.09–0.37)	< 0.001	0.10 (0.04–0.29)	< 0.001
Radiographic pattern				
Nodular bronchiectatic form	Reference		Reference	
Upper lobe cavitary form	0.45 (0.22–0.92)	0.029	0.39 (0.13–1.12)	0.079
Pulmonary function tests				
FVC (% predicted)	1.01 (0.99–1.03)	0.293		
FEV_1_ (% predicted)	1.00 (0.98–1.01)	0.742		
FEV_1_/FVC (%)	0.97 (0.94–1.01)	0.101		
Maintenance of antibiotics (≥6mo)				
M only	Reference		Reference	
M + E	9.68 (3.28–28.58)	< 0.001	18.06 (3.67–88.92)	< 0.001
M + R	4.54 (1.48–13.96)	0.008	15.82 (2.38–105.33)	0.004
M + E + R	14.91 (5.49–40.47)	< 0.001	17.12 (3.93–74.60)	< 0.001

Culture conversion was defined as at least three consecutive negative results for sputum culture the start of treatment

*Abbreviations*: *OR* odds ratio, *CI* confidence interval, *BMI* body mass index, *MAC Mycobacterium avium* complex, *FVC* forced vital capacity, *FEV*_*1*_ forced expiratory volume in 1 s, *M* macrolide, *E* ethambutol, *R* rifampicin

Predictors for a microbiological cure as well as treatment failure were also evaluated. Maintenance of both ethambutol and rifampicin (adjusted OR 5.12 with 95% CI 1.72–15.24), or maintenance of ethambutol (adjusted OR 5.74 with 95% CI 1.54–21.42) with macrolide was associated with higher rates of microbiological cure, while maintenance of rifampicin with macrolide was not (adjusted OR 2.43 with 95% CI 0.69–8.58) (Table 5). Compared with the macrolide only group, odds of treatment failure decreased when ethambutol and rifampicin were maintained with macrolide (adjusted OR 0.09 with 95% CI 0.01–0.53). However, maintenance of ethambutol (adjusted OR 0.17 with 95% CI 0.03–1.09) or rifampicin (adjusted OR 0.13 with 95% CI 0.01–1.13) with macrolide did not have this effect (Table 6).

**Table 5:** Predictors for microbiological cure among patients who completed treatment

Variables	Unadjusted OR (95% CI)	*P*	Adjusted OR (95% CI)	*P*
Age (years)	0.96 (0.93–1.00)	0.029	0.99 (0.96–1.03)	0.615
BMI (kg/m^2^)	1.13 (0.97–1.32)	0.112		
Sex, female	1.50 (0.76–2.96)	0.238		
History of tuberculosis	0.51 (0.24–1.04)	0.065		
MAC species				
*M. avium*	Reference		Reference	
*M. intracellulare*	0.32 (0.16–0.64)	0.001	0.33 (0.15–0.73)	0.006
Smear positivity at diagnosis	0.39 (0.19–0.79)	0.008	0.46 (0.21–1.02)	0.056
Radiographic pattern				
Nodular bronchiectatic form	Reference			
Upper lobe cavitary form	0.87 (0.38–1.98)	0.738		
Pulmonary function tests				
FVC (% predicted)	1.01 (0.99–1.03)	0.229		
FEV_1_ (% predicted)	1.00 (0.99–1.02)	0.801		
FEV_1_/FVC (%)	0.97 (0.94–1.01)	0.126		
Maintenance of antibiotics (≥6mo)				
M only	Reference		Reference	
M + E	7.58 (2.19–26.26)	0.001	5.74 (1.54–21.42)	0.009
M + R	2.98 (0.93–9.57)	0.067	2.43 (0.69–8.58)	0.168
M + E + R	7.58 (2.77–20.79)	< 0.001	5.12 (1.72–15.24)	0.003

Microbiological cure was defined as maintenance of negative culture conversion at the end of treatment. Assessed among 177 patients who completed treatment

*Abbreviations*: *OR* odds ratio, *BMI* body mass index, *MAC Mycobacterium avium* complex, *FVC* forced vital capacity, *FEV*_*1*_ forced expiratory volume in 1 s, *M* macrolide, *E* ethambutol, *R* rifampicin

**Table 6:** Predictors for treatment failure among patients with ≥12 months of antimycobacterial treatment

Variables	Unadjusted OR (95% CI)	*P*	Adjusted OR (95% CI)	*P*
Age (years)	1.03 (1.00–1.06)	0.029	0.99 (0.93–1.04)	0.628
BMI (kg/m^2^)	0.86 (0.75–0.99)	0.037	0.88 (0.72–1.07)	0.194
Sex, female	0.55 (0.30–1.00)	0.048	0.76 (0.27–2.14)	0.602
History of tuberculosis	1.70 (0.90–3.23)	0.103		
MAC species				
*M. avium*	Reference		Reference	
*M. intracellulare*	2.91 (1.58–5.39)	0.001	1.46 (0.51–4.17)	0.484
Smear positivity at diagnosis	3.32 (1.79–6.16)	< 0.001	7.42 (2.68–20.56)	< 0.001
Radiographic pattern				
Nodular bronchiectatic form	Reference			
Upper lobe cavitary form	1.78 (0.89–3.58)	0.105		
Pulmonary function tests				
FVC (% predicted)	0.98 (0.97–1.00)	0.093		
FEV_1_ (% predicted)	1.00 (0.98–1.01)	0.760		
FEV_1_/FVC (%)	1.03 (1.00–1.07)	0.043	1.04 (0.99–1.09)	0.099
Maintenance of antibiotics (≥6mo)				
M only	Reference		Reference	
M + E	0.28 (0.09–0.82)	0.021	0.17 (0.03–1.09)	0.061
M + R	0.57 (0.18–1.82)	0.344	0.13 (0.01–1.13)	0.064
M + E + R	0.19 (0.07–0.50)	0.001	0.09 (0.01–0.53)	0.008

Treatment failure was defined as re-emergence of multiple positive cultures or persistence with the causative species from respiratory samples after ≥12 months of antimycobacterial treatment, while the patient is still on treatment. Assessed among 204 patients with ≥12 months of antimycobacterial treatment

*Abbreviations*: *OR* odds ratio, *BMI* body mass index, *MAC Mycobacterium avium* complex, *FVC* forced vital capacity, *FEV*_*1*_ forced expiratory volume in 1 s, *M* macrolide, *E* ethambutol, *R* rifampicin

Maintenance of both ethambutol and rifampicin or one of these with macrolide did not decrease the probability of recurrence (log-rank *p* = 0.511).

### Acquisition of macrolide resistance

Drug susceptibility testing was repeated for 47 of 66 patients for whom treatment had failed, and for 12 of 16 patients in whom MAC-PD recurred. Macrolide resistance (MIC ≥32 μg/mL) was acquired by five patients who experienced treatment failure but these did not include any with recurrence of MAC-PD. The patients who acquired macrolide resistance included three males and two females, with a median age of 59 (IQR: 58–59). *M. intracellulare* was the dominant species (four patients), and an upper lobe cavitary pattern (three patients) and positive smear results at the initiation of treatment (three patients) were also common. None could maintain the three-drug regimen: two maintained macrolide only, two maintained macrolide with ethambutol, and one maintained macrolide with rifampicin. Common causes for omitting or stopping medication included decreased visual acuity (four patients), drug-drug interactions (two patients), and severe nausea (two patients). Detailed information about each patient is provided in (Additional file 1: Table S2).

### Adverse events

During treatment, 116 (49.0%) patients experienced adverse events. Deterioration of visual acuity was the most common event reported (31.6%). Other events included elevated hepatic transaminase (5.9%), general weakness (5.5%), anorexia (5.1%), nausea (4.6%), rash (4.2%), and worsened hearing loss (3.4%).

Among the 224 patients who received ethambutol, 92 (41.1%) of them stopped taking this drug because of adverse events including deteriorated visual acuity (72 patients), anorexia (8 patients), rash (8 patients), and hepatic dysfunction (8 patients). Early cessation (< 6 months) of ethambutol was observed in 44 patients (19.6%), mainly because of deteriorated visual acuity (35 patients), rash (6 patients), and anorexia (5 patients). Of the 179 patients who received rifampicin, 47 (26.3%) of them had to stop administration due to general weakness (10 patients), nausea (9 patients), anorexia (8 patients), hepatic dysfunction (7 patients), and abdominal pain (6 patients). Early cessation (< 6 months) of rifampicin was observed in 25 patients (14.0%), mostly as a result of anorexia (6 patients), general weakness (6 patients), nausea (5 patients), hepatic dysfunction (5 patients), and rash (4 patients). Patient characteristics regarding the usage of ethambutol and rifampicin and the reasons for withholding these drugs are provided in (Additional file 1: Table S3).

## Discussion

In this study, among the 237 patients who started treatment for MAC-PD, 190 (80.2%) reached culture conversion, and 129 of the 177 patients who completed treatment (72.9%) achieved microbiological cure. Of the 204 patients who underwent ≥12 months of treatment, 66 (32.4%) were classified as having treatment failure. Sixteen of the 129 patients with microbiological cure (12.4%) experienced recurrence of MAC-PD. Most patients started out using macrolide (100.0%), ethambutol (94.5%), and rifampicin (75.9%) as a multidrug combination treatment. Maintenance (≥6 months) of both ethambutol and rifampicin with macrolide was associated with the most favorable outcomes: higher odds of culture conversion as well as microbiological cure, and lower odds of treatment failure.

Treatment outcomes in this study were comparable with those of previous studies. In two retrospective analyses and one randomized controlled trial, rates of culture conversion for MAC-PD using a macrolide-containing regimen was reported as 40–95% [14, 15, 17]. In addition, two meta-analyses reported culture conversion rates of 55–57% without recurrence [6, 8].

Our study showed that maintenance of ethambutol was more strongly associated with microbiological cure than rifampicin. Ethambutol inhibits arabinosyltransferase and blocks arabinogalactan synthesis, which forms part of the mycobacterial wall [18–20]. With such mechanism, the synergistic effect of ethambutol is expected when used with other antimycobacterial agents. The effects of ethambutol on MAC was reported in animal studies, which reduced MAC growth in the blood, liver, and spleen [21]. In a human study, ethambutol reduced the mycobacteremia level in AIDS patients [22]. Another study reported that more than 5 months of ethambutol use was associated with improvement in MAC culture [23]; however, that study used the reduction in colony count as an outcome rather than the strict criteria of culture conversion or treatment success [16, 24]. Given the favorable effects of maintenance of ethambutol in our study, cessation of ethambutol early during the course of treatment based on uncertain adverse events should be avoided [25, 26].

Although our study raised concerns regarding the effectiveness of rifampicin and macrolide without ethambutol in terms of microbiological cure, the importance of a rifamycin in the treatment of MAC was noted in earlier studies: rifabutin was effective in reducing mycobacterial titers among AIDS patients with disseminated MAC infection [27], and synergistic effects of rifampicin and ethambutol on MAC were shown in vitro in liquid media [28]. In addition, maintenance of a single drug (rifampicin or ethambutol) supplemented with macrolide could result in the emergence of clarithromycin resistance, as was shown in the present study. However, the effect of rifampicin has been questioned in other studies: in an open-label randomized controlled trial comparing a two-drug regimen (clarithromycin, ethambutol) with a three-drug regimen (clarithromycin, ethambutol, and rifampicin), the 12-month sputum-negative conversion rates between the two groups were equivalent [17]. Another study demonstrated a good treatment success rate among MAC-PD patients treated with a three-drug regimen of macrolide, ethambutol, and clofazimine without rifampicin [29]. The weaker effect of rifampicin for the treatment of MAC-PD could be explained based on a lowered serum level of macrolide through induction of cytochrome p450 by rifampicin, although one previous report suggested that the serum concentration does not affect treatment outcomes [30, 31]. It can also be explained that rifampicin has antimycobacterial activity by binding to the β subunit of RNA polymerase, thereby inhibiting RNA synthesis [32]. In slowly growing mycobacteria such as MAC species, such a mechanism may result in a bacteriostatic effect rather than a bactericidal effect [33]. In fact, clinical significance of synergistic activity of rifampicin and ethambutol against slow-growing NTM was doubted in a previous study [34].

Although maintenance of ethambutol appears to be very important in the treatment of MAC-PD, the possibility of optic neuropathy should be considered with prolonged use of ethambutol. Among patients taking ethambutol for pulmonary TB, ethambutol-induced optic neuropathy develops in 1–2% in a dose-dependent manner [35]. In addition, about 0.2–0.3% of patients may experience irreversible visual function loss. In our study, about one-third of patients experienced worsened visual acuity and it was major cause of earlier cessation of ethambutol. Given its effectiveness as well as risk of optic neuropathy, the best strategy could be long-term use of ethambutol with active surveillance of visual acuity and rapid objective assessment in the setting of subjective deterioration.

This study had some limitations. First, it was a retrospective study based on a single center. Patients receiving treatment at the time of data retrieval could not be included in several of the analyses. Potential confounders that were not considered in our study (e.g. concomitant medications or the radiographic extent of the disease) may exist between the study groups. Future well-designed, randomized controlled clinical trials will be necessary to fully understand the roles of ethambutol and rifampicin in MAC-PD. Second, the adverse events of medication were self-reported, and the visual acuity loss was not evaluated objectively. This may have led to a high prevalence of ethambutol-induced ocular toxicity. However, there are currently no standardized protocols for detecting ethambutol-induced ocular toxicity, and self-reported visual loss is a reliable means to determine optic neuritis from ethambutol [36]. Third, re-infection and relapse of MAC could not be differentiated between among patients with recurrence because genetic comparisons of the causative strains were not performed in our study. The lack of such analyses might have resulted in the insignificant associations between maintenance of ethambutol or rifampicin and the risk of recurrence after microbiological cure.

## Conclusions

Maintenance (≥6 months) of ethambutol and rifampicin treatment with macrolide was associated with the most favorable treatment outcomes among patients with MAC-PD. Given the impact of ethambutol on microbiological cure, clinicians should maintain ethambutol unless definite and serious adverse events develop from ethambutol treatment.

## Supplementary information

**Additional file 1: Table S1.** Detailed dosage information about the antimycobacterial agents used in the treatment of *Mycobacterium avium* complex lung disease. **Table S2.** Detailed description of 5 patients who developed clarithromycin resistance during treatment failure. **Table S3.** Patient characteristics according to the use of ethambutol and rifampicin.

## Data Availability

Data are available from the corresponding author upon a reasonable request.

## Abbreviations

AIDS: Acquired immune deficiency syndrome
CI: Confidence interval
IQR: Interquartile range
MAC: *Mycobacterium avium* complex
MIC: Minimal inhibitory concentration
NTM: Nontuberculous mycobacteria
OR: Odds ratio
PD: Pulmonary disease
