# Supplementary material for: Role of ethambutol and rifampicin in the treatment of Mycobacterium avium complex pulmonary disease
Source: BMC Pulm Med. 2019 Nov 11;19:212. doi: 10.1186/s12890-019-0982-8 (PMC6849249; doi:10.1186/s12890-019-0982-8)
Supplement: Supplementary file 1 — Additional file 1: Table S1. Detailed dosage information about the antimycobacterial agents used in the treatment of Mycobacterium avium complex lung disease. Table S2. Detailed description of 5 patients who developed clarithromycin resistance during treatment failure. Table S3. Patient characteristics according to the use of ethambutol and rifampicin. [file 12890_2019_982_MOESM1_ESM.docx]

**Table S1.** Detailed dosage information about the antimycobacterial agents used in the treatment of *Mycobacterium avium* complex lung disease

| Antimycobacterial agent | Dose | Values | |
| --- | --- | --- | --- |
|  |  | Daily  regimen  n=146 | Three-times-weekly  regimen n=91 |
| Macrolide, n (%) |  |  |  |
| Azithromycin |  |  |  |
|  | 250mg | 90 (61.6%) | 12 (13.2%) |
|  | 500mg | 3 (2.1%) | 78 (85.7%) |
| Clarithromycin |  |  |  |
|  | 500mg | 2 (1.4%) | - |
|  | 1000mg | 51 (34.9%) | 1 (1.1%) |
| Ethambutol (mg/kg), median (IQR) |  | 15.4 (13.8-17.0) | 22.6 (19.4-24.1) |
| Rifamycin, n (%) |  |  |  |
| Rifampicin |  |  |  |
|  | 450mg | 29 (19.9%) | 1 (1.1%) |
|  | 600mg | 74 (50.7%) | 72 (79.1%) |
|  | 1200mg |  | 1(1.1%) |
| Rifabutin |  |  |  |
|  | 300mg | 1 (0.7%) | - |

Values are presented as numbers (%) or median (interquartile range).

**Table S2.** Detailed description of 5 patients who developed clarithromycin resistance during treatment failure

| Patient no | Patient 1 | Patient 2 | Patient 3 | Patient 4 | Patient 5 |
| --- | --- | --- | --- | --- | --- |
| Age | 59 | 70 | 58 | 59 | 52 |
| Sex | Male | Male | Female | Male | Female |
| Smoking | Never | Ex-smoker | Never | Ex-smoker | Never |
| History of Tuberculosis | Yes | No | No | No | Yes |
| MAC species | intracellulare | intracellulare | avium | intracellulare | intracellulare |
| Smear positivity at diagnosis | No | No | Yes | Yes | Yes |
| Radiographic pattern | Upper lobe cavitary type | Upper lobe cavitary type | Nodular bronchiectatic type | Upper lobe cavitary type | Nodular bronchiectatic type |
| Initial MIC of clarithromycin | 1 µg/mL | 4 µg/mL | 4 µg/mL | 2 µg/mL | 2 µg/mL |
| Treatment regimen | M+E+Q+Inj | M+E+R | M+E+Clo+Inj | M+E+Clo+Q | M+R+Clo+Inj |
| Treatment duration | 47.2 months | 13.8 months | 18.4 months | 21.9 months | 20.5 months |
| Maintenance of major agent | M | M | M+E | M+E | M+R |
| Reasons for omitting/stopping medication | Visual discomfort, Drug-induced depression | Visual discomfort, Nausea | Visual discomfort, Drug-to-drug interaction | Nausea,  Drug-to-drug interaction | Visual discomfort, General weakness |

Abbreviations: MAC, *Mycobacterium avium* complex; MIC, minimal inhibitory concentration; M, macrolide; E, ethambutol; Q, fluoroquinolone; Inj, injectable agent; R, rifampicin; Clo, clofazimine.

**Table S3.** Patient characteristics according to the use of ethambutol and rifampicin

| Variables | Patients who did not receive or stopped ethambutol  at any period  n=107 | Patients who did not receive or stopped rifampicin  at any period  n=107 | Patients who received and continued ethambutol but not rifampicin  n=56 | Patients who received and continued rifampicin but not ethambutol  n=52 |
| --- | --- | --- | --- | --- |
| Age (years) | 68 (58–74) | 68 (58–75) | 65 (57–75) | 65 (57–73) |
| BMI (kg/m^2^) | 20.3 (19.0–22.1) | 20.0 (18.4–22.1) | 20.6 (18.1–22.2) | 20.3 (19.0–22.7) |
| Sex, female | 62 (57.9%) | 60 (56.1%) | 34 (60.7%) | 34 (65.4%) |
| Smoking history |  |  |  |  |
| Never smoker | 69 (64.5%) | 63 (58.9%) | 32 (57.1%) | 38 (82.6%) |
| Former smoker | 22 (20.6%) | 26 (24.3%) | 13 (23.2%) | 7 (15.2%) |
| Current smoker | 6 (5.6%) | 6 (5.6%) | 3 (5.4%) | 1 (2.2%) |
| Comorbidities |  |  |  |  |
| Asthma | 4 (3.7%) | 2 (1.9%) | 0 (0.0%) | 2 (3.9%) |
| COPD | 2 (1.9%) | 2 (1.9%) | 0 (0.0%) | 0 (0.0%) |
| History of tuberculosis | 31 (29.0%) | 33 (30.9%) | 15 (26.8%) | 10 (19.2%) |
| MAC species |  |  |  |  |
| *M. avium* | 47 (43.9%) | 48 (44.9%) | 27 (48.2%) | 24 (46.2%) |
| *M. intracellulare* | 59 (55.1%) | 57 (53.3%) | 28 (50.0%) | 28 (53.9%) |
| *M. chimaera* | 1 (0.9%) | 2 (1.9%) | 1 (1.8%) | 0 (0.0%) |
| Smear positivity at diagnosis | 42 (39.3%) | 42 (39.3%) | 16 (28.6%) | 5 (35.7%) |
| Radiographic pattern |  |  |  |  |
| Nodular bronchiectatic | 85 (79.4%) | 82 (76.6%) | 44 (78.6%) | 44 (84.6%) |
| Upper lobe cavitary | 22 (20.6%) | 25 (23.4%) | 12 (21.4%) | 8 (15.4%) |
| Pulmonary function tests |  |  |  |  |
| FVC (% predicted) | 87 (74–98) | 84 (73–95) | 85 (75–94) | 93 (78–102) |
| FEV_1_ (% predicted) | 94 (79–111) | 93 (78–103) | 92 (78–102) | 95 (78–111) |
| FEV_1_/FVC (%) | 78 (71–87) | 78 (71–85) | 75 (70–82) | 77 (70–82) |
| Reasons for stopping drug |  |  |  |  |
| Visual discomfort | 81 (75.7%) | 35 (32.7%) | 3 (5.4%) | 47 (90.4%) |
| Gastrointestinal discomfort | 16 (15.0%) | 27 (25.2%) | 14 (25.0%) | 3 (5.8%) |
| Drug-to-drug interaction | 12 (11.2%) | 26 (24.3%) | 15 (26.8%) | 0 (0.0%) |
| Hypersensitivity | 11 (10.3%) | 14 (13.1%) | 5 (8.9%) | 3 (5.8%) |
| Hepatic dysfunction | 11 (10.3%) | 14 (13.1%) | 8 (14.3%) | 0 (0.0%) |
| General weakness | 5 (4.7%) | 11 (10.3%) | 7 (12.5%) | 0 (0.0%) |

The columns are not mutually exclusive. Values are presented as number (percentage) or median (interquartile range). Abbreviations: BMI, body mass index; COPD, chronic obstructive pulmonary disease; MAC, *mycobacterium avium complex*; FVC, forced vital capacity; FEV_1_, forced expiratory volume in 1 second.
